# Urine Metabolomics and Machine Learning Identify Metabolic Features and Potential Biomarkers of HTLV-1-Associated Myelopathy (HAM)

**DOI:** 10.3390/ijms27041827

**Published:** 2026-02-14

**Authors:** Lorena Abreu Fernandes, Youko Nukui, Rosa Maria Marcusso, Michel Elyas Jung Haziot, Augusto César Penalva de Oliveira, Jorge Casseb, Patricia Bianca Clissa, Ana Olivia de Souza, Silas G. Villas-Boas, Sabri Saeed Sanabani

**Affiliations:** 1 Postgraduate Program in Translational Medicine, Department of Medicine, Federal University of São Paulo (UNIFESP), São Paulo 04023-062, Brazil; lafernandes@unifesp.br; 2 Department of Hematology, Faculty of Medicine, University of São Paulo, São Paulo 05403-000, Brazil; youko.nukui@hc.fm.usp.br; 3 Department of Neurology, Emilio Ribas Institute of Infectious Diseases, São Paulo 01246-900, Brazil; rosa.marcusso@emilioribas.sp.gov.br (R.M.M.); michel.haziot@emilioribas.sp.gov.br (M.E.J.H.); augusto.oliveira@emilioribas.sp.gov.br (A.C.P.d.O.); 4 Laboratory of Medical Investigation LIM-56, Department of Dermatology, University of São Paulo, São Paulo 05403-000, Brazil; jcasseb@usp.br; 5 Immunopathology Laboratory, Butantan Institute, São Paulo 05503-900, Brazil; patricia.clissa@butantan.gov.br; 6 Development and Innovation Laboratory, Instituto Butantan, Avenida Vital Brasil, 1500, São Paulo 05503-900, Brazil; ana.souza@butantan.gov.br; 7 Luxembourg Institute of Science and Technology, 5, Avenue des Hauts-Fourneaux, L-4362 Esch-sur-Alzette, Luxembourg; silasgvboas@gmail.com; 8 Laboratory of Medical Investigation LIM-03, Department of Pathology, University of São Paulo, São Paulo 05403-000, Brazil

**Keywords:** HTLV-1, HAM/TSP, metabolomics, machine learning, biomarkers

## Abstract

Human T-cell lymphotropic virus type 1 (HTLV-1) can cause HTLV-1-associated myelopathy/tropical spastic paraparesis (HAM/TSP), a progressive neuroinflammatory disease that lacks noninvasive biomarkers. We used untargeted urine metabolomics with machine learning to profile 113 participants (39 with HAM, 17 with intermediate syndrome, 33 asymptomatic carriers, and 24 healthy controls). Gas chromatography–mass spectrometry identified 175 metabolites, 86 of which showed significant differences (fold change > 2, FDR *p* < 0.05). Multivariate analyses revealed distinct but partially overlapping metabolic profiles: sPLS-DA captured a reproducible yet moderately discriminative signal, while nonlinear machine learning models (Random Forest and SVM) achieved robust group separation, with HAM displaying a distinct metabolic signature. Key discriminators included Unknown_151, Unknown_127, histidine, alanine, and 4-hydroxyphenylacetic acid, which showed marked reductions in HAM and yielded ROC AUCs of 0.855–0.871. Pathway and disease enrichment analyses highlighted disturbances in amino acid metabolism, particularly beta-alanine and aromatic amino acids, along with disease signatures related to inherited amino acid handling disorders such as hyperlysinemia. These results demonstrate that urinary metabolomics combined with machine learning can identify potential noninvasive biomarkers for HAM and provide novel insights into HTLV-1-associated pathophysiology.

## 1. Introduction

Human T-cell lymphotropic virus type 1 (HTLV-1) is an oncogenic retrovirus that is causally linked to serious diseases, particularly adult T-cell leukemia/lymphoma (ATL) and HTLV-1-associated myelopathy/tropical spastic paraparesis (HAM/TSP), a chronic, progressive neuroinflammatory disease of the spinal cord [1,2]. The clinical diagnosis of HAM/TSP remains difficult because the neurological symptoms overlap with those of other progressive myelopathies, there are no pathognomonic imaging findings, and one must rely on laboratory evidence of HTLV-1 infection in serum and cerebrospinal fluid [3]. Although new inflammatory and neurodegeneration markers are being explored to improve diagnosis and monitoring, there are currently no validated noninvasive biomarkers for early detection or reliable prediction of disease progression [4,5,6]. This diagnostic uncertainty delays intervention and hinders effective care and treatment for affected individuals [3].

Metabolomics, which provides comprehensive profiles of small molecule metabolites in biological fluids, offers a promising approach for the identification of disease-specific metabolic signatures and biomarker candidates [7,8]. Previous studies on neuroinfectious and neuroinflammatory diseases have shown that metabolomic changes reflect underlying pathophysiological changes and may even precede clinical manifestations [9,10,11]. In particular, urine, an easily accessible biofluid, has proven to be a suitable matrix for the non-invasive discovery of metabolic biomarkers [12,13].

Recent advances in high-resolution mass spectrometry and bioinformatic platforms now enable global (untargeted) analysis of urinary metabolites [14]. However, the vast and complex datasets generated require sophisticated analysis tools. The integration of machine learning (ML) with metabolomic data has yielded significant improvements in disease stratification, biomarker discovery, and predictive model accuracy for a range of conditions, including infectious diseases [15,16,17,18,19]. By leveraging algorithms such as random forests, logistic regression, and support vector machines, ML can identify subtle yet robust metabolic differences among patient cohorts and highlight candidate biomarkers with translational potential [20,21].

To date, no study has systematically investigated the urinary metabolome of HTLV-1-infected HAM patients, nor have machine learning-based approaches been used to discover biomarkers in this population. This knowledge gap limits the development of non-invasive diagnostic tools and a deeper understanding of the metabolic changes associated with HTLV-1 neuroinflammatory complications. Therefore, this study represents the first comprehensive attempt to characterize the urinary metabolic landscape across the clinical status of HTLV-1 infection and apply advanced machine learning techniques to identify discriminative metabolic features and novel biomarkers in urine for the early detection and classification of HTLV-1-associated myelopathy.

## 2. Results

### 2.1. Characteristics of Study Population

The study cohort consisted of 113 participants classified into four groups: 39 HAM patients, 17 IS patients, 33 ASC, and 24 HCs (Table 1). The HAM group had a predominantly female composition (28 females, 11 males) with a median age of 57 years. Urinary dysfunction was common in the HAM group, with 15 experiencing urine retention, 22 with urinary incontinence, and 8 presenting both symptoms. Clinical history showed variability in diagnosis duration, with most patients living with HAM for over 10 years. The majority of HAM patients (25/39) were undergoing treatment, predominantly with oral corticosteroids or pulse therapy. The IS group comprised 17 participants (15 females, 2 males) with a median age similar to HAM patients (57 years). Some IS patients also exhibited urinary symptoms: 4 with urine retention, 7 with incontinence, and 2 with both conditions. Diagnostic and treatment data for the IS group were not available at this stage. The ASC group shared a female predominance (25 females, 8 males) and were younger, with a median age of 60 years, and remained asymptomatic carriers. The HC group had balanced sex distribution and a median age of 39 years.

### 2.2. Global Metabolomics Analysis and Statistical Approaches

**Figure 1:**
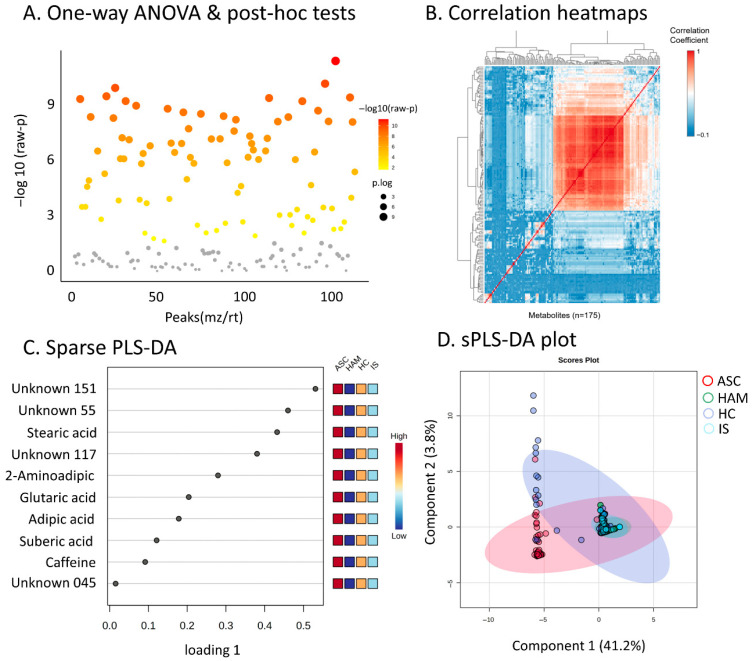
Global metabolomics analysis and multivariate statistics. (**A**) Scatter plot of one-way ANOVA *p*-values, showing −log_10_ (raw *p*) for 175 metabolite peaks (*m*/*z*–retention time) across HAM, IS, ASC, and HC groups; gray points denote non-significant features, whereas yellow-orange-red points indicate progressively lower *p*-values. (**B**) Metabolite–metabolite correlation heatmap (n = 175) with hierarchical clustering, illustrating positively (red) and negatively (blue) correlated clusters consistent with co-regulated metabolic modules. (**C**) Sparse PLS-DA loading plot for Component 1, displaying the contribution (loading weights) of selected metabolites (e.g., Unknown 151, Unknown 55, stearic acid, 2-aminoadipic acid) to group discrimination; the adjacent red–white–blue strip depicts relative abundance patterns (high to low) of these metabolites across the four clinical groups. (**D**) sPLS-DAS scores plot of Components 1 (41.2% explained variance) and 2 (3.8%), showing sample distribution by group (ASC, HAM, HC, IS) with 95% confidence ellipses; HAM samples form a more distinct cluster, whereas ASC, HC, and IS exhibit partial overlap.

The untargeted metabolomics analysis of urine samples identified a total of 175 metabolites, comprising 124 known and 51 unknown compounds. Statistical evaluation using one-way ANOVA followed by Tukey’s post hoc tests revealed 86 metabolites with evidence of differences across the four study groups based on a fold change greater than 2 and FDR-adjusted *p*-values below 0.05. These metabolites are visualized in the scatter plot (Figure 1A), with more significant features represented by increased point size and warmer colors. Hierarchical clustering of metabolite correlations across the complete dataset is presented in the correlation heatmap (Figure 1B). This heatmap suggests clusters of positively and negatively correlated metabolites that may reflect co-regulated metabolic networks or pathways associated with the different clinical groups, including the IS group, thereby expanding the metabolic landscape associated with the HTLV 1 infection spectrum. To explore metabolites that contribute most to discrimination among HAM, ASC, HC, and IS, we applied sparse Partial Least Squares Discriminant Analysis (sPLS DA). The loadings plot for the first component (Figure 1C) highlights metabolites such as stearic acid, glutaric acid, adipic acid, suberic acid, caffeine, and several unknowns, each displaying distinct abundance trends across the four groups, which are summarized by the adjacent heatmaps. The sPLS DA scores plot based on the first two components (Figure 1D) illustrates partial separation of the clinical groups: HAM samples tend to cluster on one side of Component 1, whereas ASC, HC, and IS show broader overlap, with IS samples occupying an intermediate region between HAM and ASC. Confidence ellipses illustrate within-group variability and inter-group overlap, indicating that urinary metabolomic profiles differentiate HAM to some extent, while ASC, HC, and IS exhibit more gradual differences rather than sharp boundaries. Model validation using 10-fold cross-validation and permutation testing (n = 100) indicated that a three-component PLS DA model (R^2^ = 0.44, Q^2^ = 0.23, permutation *p* < 0.01) captured a reproducible but moderate discriminative signal. In view of the limited predictive power of this linear model, and recognizing the complexity of clinical urine metabolomics data, we subsequently focused on non-linear supervised methods (Random Forest and SVM), which provided higher classification performance (AUC > 0.85) and better captured the multivariate metabolic signature associated with HAM.

### 2.3. Global Assessment of Confounding Factors

To ensure that the identified metabolic signatures were specific to HAM pathology and not influenced by demographic differences, such as the age difference between ASC and HAM groups, we conducted a global sensitivity analysis using General Linear Models (GLM). We compared the statistical significance of all 175 metabolites before and after adjusting for age and sex as covariates. As shown in Figure S1, the adjusted *p*-values were almost perfectly correlated with the unadjusted *p*-values, indicating that demographic factors had a negligible effect on the metabolic profile. Specifically, 93% (80/86) of the differentially expressed metabolites, including all top discriminative biomarkers such as histidine and beta-alanine, remained statistically significant after adjustment, confirming the robustness of these findings.

### 2.4. Key Discriminative Metabolites Identified by Multivariate and ROC Analyses

**Figure 2:**
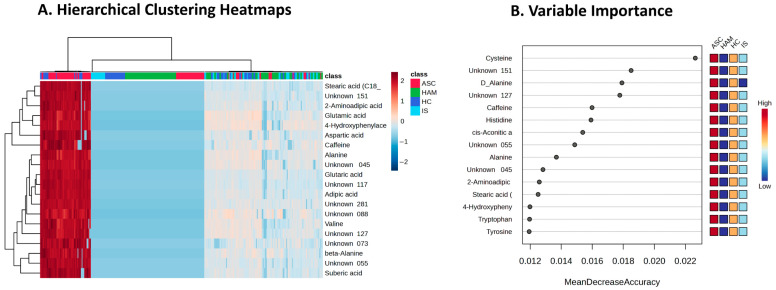
Key discriminative metabolites identified by multivariate analyses. (**A**) Hierarchical clustering heatmap of the top 20 metabolites across HAM, IS, ASC, and HC groups, with rows clustered by metabolite similarity and columns by sample; abundance levels range from low (blue) to high (red), revealing group-specific patterns (top bar: ASC red, HAM green, HC blue, IS cyan). (**B**) Random Forest variable importance ranking (500 trees, 25 predictors, OOB error = 0.286), plotting mean decrease in accuracy for top metabolites (e.g., cysteine, Unknown 151, D-alanine); adjacent heatmap shows relative abundances (low: blue; high: red) across groups.

To investigate metabolite patterns that differentiate the study groups, we performed hierarchical clustering of the top 20 discriminative metabolites and assessed their discriminatory contribution using random forest analysis. This approach allowed us to visualize group-specific abundance profiles and to rank metabolites according to their impact on classification accuracy. Figure 2A shows a hierarchical clustering heatmap of the top 20 metabolites, revealing distinct expression patterns across the four study groups (ASC, HAM, HC, and IS). Metabolites such as stearic acid, 2-aminoadipic acid, glutamic acid and several unidentified compounds display clear differences in abundance, with clusters of metabolites tending to show higher or lower levels in particular groups. Figure 2B presents the random forest variable-importance ranking (500 trees, 25 predictors, OOB error = 0.286), in which metabolites including cysteine, Unknown_151, D-alanine, Unknown_127, caffeine and histidine exhibit the highest mean decrease in accuracy values, indicating a strong contribution to correct group classification. Most of these top metabolites showed lower concentrations in HAM patients than in the other groups, a trend that is reflected in the heatmap adjacent to the variable-importance plot. This overall reduction in several key metabolites may reflect metabolic disturbances related to the pathophysiology of HAM. Collectively, these machine-learning analyses support the existence of a robust biosignature composed largely of altered amino acids, such as histidine and alanine, that serve as informative indicators for distinguishing HAM patients from other HTLV-1-infected individuals.

**Figure 3:**
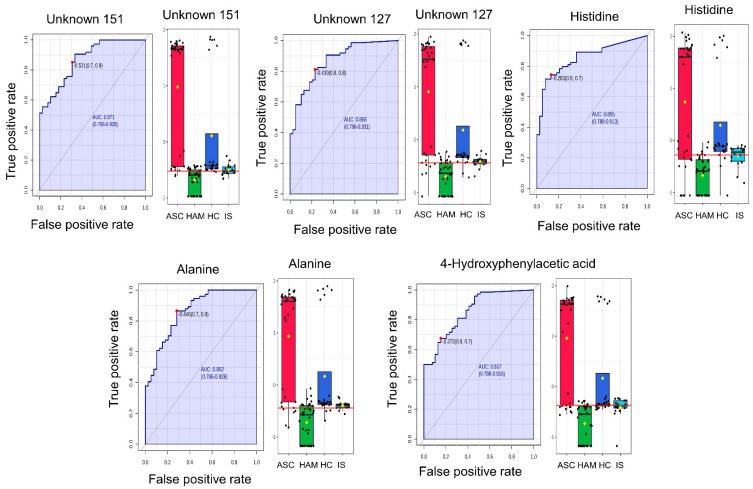
Diagnostic performance of key metabolites distinguishing HAM from non-HAM groups (ASC, IS, HC). For each metabolite (Unknown 151, Unknown 127, histidine, alanine, 4-hydroxyphenylacetic acid), the left panel displays the receiver operating characteristic (ROC) curve, plotting true positive rate versus false positive rate for HAM classification, with the area under the curve (AUC) and corresponding 95% confidence interval indicated. The right panel shows group-wise boxplots of normalized metabolite abundances for ASC, HAM, HC, and IS (colors as shown in the figure), with individual data points overlaid, illustrating distinct distribution patterns between HAM and non-HAM subjects.

Building on these multivariate results, we next assessed the individual diagnostic performance of key metabolites using ROC curve analysis to determine their ability to discriminate HAM patients from all other groups. ROC curve analysis depicted in Figure 3 identified five metabolites namely Unknown_151 (AUC = 0.870, *p* = 1.05 × 10^−4^, log_2_FC = −20.007), Unknown_127, histidine, alanine, and 4-hydroxyphenylacetic acid, as the most accurate in distinguishing HAM patients from all other groups. The Unknown_151 acts as a distinct “metabolic feature”. All five showed excellent discriminatory performance, with AUROC values ranging from 0.855 to 0.871, indicating high sensitivity and specificity under the selected cutoff criteria. For the HAM versus non-HAM comparison, we also examined class-specific sensitivity and specificity at these cutoffs, confirming that HAM detection performance was consistent with the high AUC values and was not solely influenced by the larger size of the non-HAM group. The boxplots reveal a consistent trend across these metabolites, with markedly reduced concentrations in HAM patients compared to ASC, HC, and IS groups. In contrast, ASC individuals displayed the highest metabolite abundances, followed by intermediate or low levels in HC and IS. This clear separation in metabolite levels between HAM and non-HAM groups, coupled with the strong ROC performance, highlights their potential as robust biomarkers for HAM.

**Figure 4:**
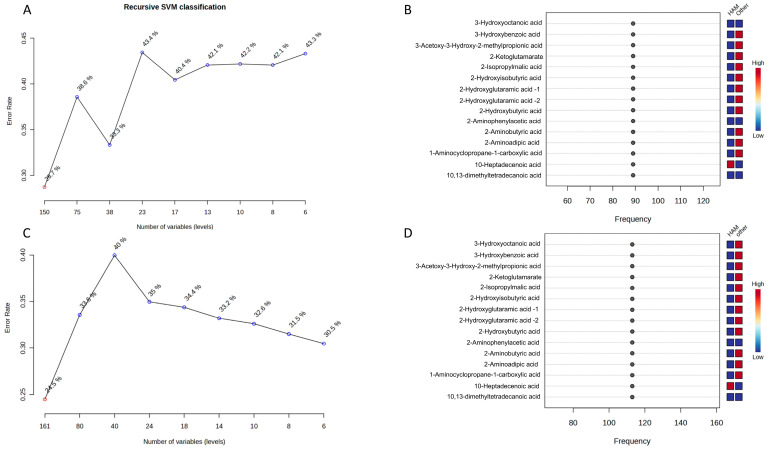
Recursive SVM feature selection and variable importance for distinguishing HAM from non-HAM subjects. (**A**) Recursive SVM classification curve for HAM versus ASC + IS, showing cross-validated error rate as a function of the number of variables retained in the model; each point represents a linear SVM classifier trained on a decreasing subset of metabolites. (**B**) SVM variable-importance plot for HAM versus ASC + IS, ranking the top features by their selection frequency (%) among the best classifiers; adjacent red–white–blue tiles indicate relative abundances (high to low) in HAM and in the combined ASC + IS group. (**C**) Recursive SVM classification curve for HAM versus ASC + IS + HC, illustrating a progressive reduction in error rate as the number of variables decreases, with the lowest error observed for the smallest subset of six metabolites. (**D**) SVM variable-importance plot for HAM versus ASC + IS + HC, displaying the most frequently selected metabolites and their relative concentration profiles in HAM compared with the aggregated non-HAM group, highlighting a compact biomarker panel that supports accurate discrimination.

After identifying the differential metabolites, we used a support vector machine (SVM)-based recursive feature selection strategy in MetaboAnalyst 6.0 to assess their combined biomarker potential for distinguishing HAM patients from other groups. In the initial analysis comparing HAM with the combined ASC + IS group (Figure 4A,B), the recursive SVM classification curve showed that cross-validated error rates remained relatively high (around 40%) as the number of variables decreased (Figure 4A). This suggests that separating HAM from clinically related non-HAM patients requires a broader set of metabolites rather than a minimal panel. Consistent with this, the associated SVM variable-importance profile ranked several 3-hydroxy fatty acids, aromatic acids, and amino acid derivatives among the most frequently selected features, with clear concentration differences between HAM and ASC + IS (Figure 4B). When we expanded the comparison to HAM versus all non-HAM subjects (ASC + IS + HC), the classifier’s behavior changed: the error rate progressively decreased as features were removed, reaching a minimum of approximately 30% with only six variables (Figure 4C). This demonstrates that a compact metabolite panel can accurately discriminate HAM in this broader context. The corresponding importance plot highlighted largely overlapping metabolite classes, again showing pronounced shifts between HAM and non-HAM (Figure 4D). These findings complement the univariate ROC results by indicating that, beyond individual markers, a small SVM-selected subset of metabolites captures a robust multivariate signature capable of distinguishing HAM patients from both symptomatic non-HAM individuals and healthy controls. Notably, some strong individual biomarkers, such as alanine, were not always selected in the final compact SVM panel, highlighting that different algorithms can capture complementary aspects of the HAM-associated metabolic signature. To define our candidate biomarker set, we focused on metabolites that were repeatedly prioritized across univariate analysis (FDR significant with large effect sizes), RF variable importance, and SVM-based recursive feature selection, emphasizing features that were consistently informative across methods rather than relying on a single algorithm.

### 2.5. Pathway and Disease Signature Enrichment Analysis

**Figure 5:**
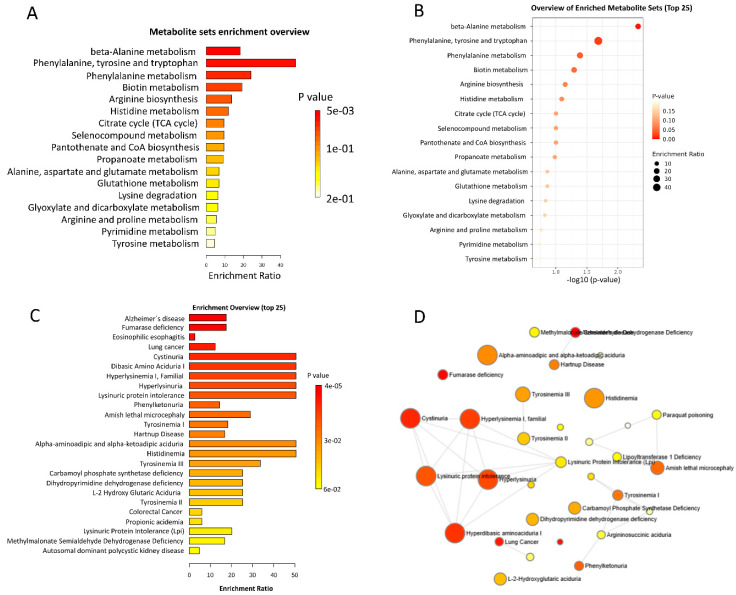
Pathway and disease signature enrichment analysis based on top discriminatory metabolites across HAM, IS, ASC, and HC groups. (**A**) Metabolite sets enrichment overview as a bar chart, ranking pathways (e.g., phenylalanine, tyrosine, tryptophan biosynthesis) by enrichment ratio (0–40+), color-coded by *p*-value (red: low ~5 × 10^−3^; yellow: high ~2 × 10^−1^). (**B**) Overview of top 25 enriched metabolite sets in a scatter plot, with x-axis −log10(*p*-value) (0–2.0), pathways labeled, dots sized and colored by enrichment ratio (small black: ~10; large red: ~40). (**C**) Disease signatures enrichment overview (top 25) as a bar chart, ranking signatures (e.g., hyperdibasic aminoaciduria I) by enrichment ratio (0–50+), color-coded by *p*-value (red: low ~4 × 10^−5^; yellow: high ~6 × 10^−2^). (**D**) Disease network visualization with interconnected nodes sized and colored by enrichment strength (large red: high, e.g., hyperdibasic aminoaciduria I; small yellow: low), illustrating clusters around amino acid metabolism disorders.

Building upon the identification of key discriminatory metabolites with strong biomarker potential, we further explored the broader biological context of these metabolic alterations through pathway and disease signature enrichment analyses. This investigation aimed to highlight metabolic pathways and disease-related processes that appear most affected across the HAM, IS, ASC, and HC groups, providing additional insight into the underlying pathophysiology as detailed in Figure 5. Briefly, the results present a comprehensive pathway and disease signature enrichment analysis based on the top significant metabolites discriminating HAM, IS, ASC, and reference groups. The metabolite set enrichment overview (Figure 5A) indicates marked enrichment of amino acid biosynthesis and metabolism pathways, notably phenylalanine, tyrosine, and tryptophan biosynthesis, which shows one of the highest enrichment ratios and a very low *p*-value. Beta-alanine metabolism and phenylalanine metabolism pathways also display prominent enrichment, suggesting pronounced alterations in amino acid–related metabolic processes. Other pathways such as biotin metabolism, arginine biosynthesis, histidine metabolism, citrate (TCA) cycle, and glutamate metabolism show moderate enrichment, pointing to broader metabolic changes involving energy metabolism and amino acid cycling. The detailed pathway list in Figure 5B is consistent with these observations, emphasizing the central involvement of amino acid metabolism, including beta-alanine, phenylalanine, tyrosine, and tryptophan pathways, together with key energy and cofactor biosynthesis routes.

The disease signatures enrichment overview (Figure 5C) further supports the metabolic relevance of these perturbations, with disorders such as hyperdibasic aminoaciduria I and familial hyperlysinemia I showing high enrichment ratios and very low *p*-values, suggesting a close resemblance between the observed metabolic alterations and known defects in amino acid metabolism and renal amino acid handling. Additional enriched signatures, including lysinuric protein intolerance and phenylketonuria, also show substantial enrichment, reinforcing the involvement of lysine and phenylalanine metabolic pathways. The disease network visualization in Figure 5D depicts these enriched signatures as interconnected nodes with gradient colors and sizes reflecting enrichment strength. Nodes corresponding to conditions such as hyperdibasic aminoaciduria I and familial hyperlysinemia are among the larger and more intensely colored elements in the network, highlighting their prominence within the enriched disease space. Overall, the network clusters around amino acid metabolism disorders, suggesting a coherent pattern of metabolic alterations compatible with disturbed protein catabolism and possible renal involvement in the context of HAM and related HTLV-1 infection states, while additional signatures such as tyrosinemia, phenylketonuria, and carbamoyl phosphate synthetase deficiency appear with intermediate enrichment, outlining a broader spectrum of metabolic perturbations.

**Figure 6:**
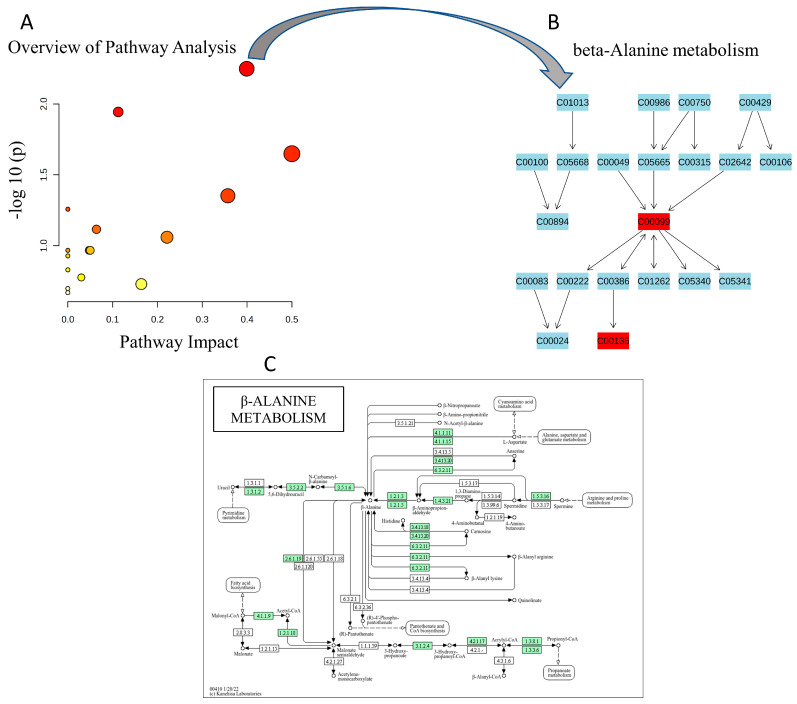
Topology-based pathway analysis of the enriched beta-alanine metabolism pathway. (**A**) Pathway impact plot as a scatter of metabolic pathways, with x-axis showing impact scores (0–0.5) and y-axis −log10(*p*-values) (0–2.0); beta-alanine metabolism highlighted as a large red dot (~0.4 impact, 1.56 −log(*p*)), others as smaller orange/yellow dots. (**B**) Metabolite network diagram depicting hierarchical topology with KEGG IDs and directed arrows for reaction flows; beta-alanine (C00099, red) central, branching to upstream (e.g., C00113, C00986) and downstream (e.g., C00894, C00386) compounds, L-histidine (C00135, red) noted. (**C**) Detailed biochemical schematic of beta-alanine metabolism, illustrating sources (pyrimidine catabolism, polyamine degradation, aspartate decarboxylation) and conversions (to pantothenate/CoA, dipeptides like carnosine/anserine), with enzyme EC numbers and integrations to related pathways (arginine/proline/glutamate metabolism).

Complementing the enrichment analyses presented in Figure 5, a detailed topology-based pathway analysis was conducted on the significantly enriched beta-alanine metabolism pathway, as illustrated in Figure 6. The pathway impact plot (Figure 6A) displays a scatter of dots representing various metabolic pathways, with the x-axis denoting pathway impact scores ranging from 0 to 0.5 and the y-axis showing −log10(*p*-values) from 0 to 2.0; the beta-alanine metabolism pathway is highlighted as a prominent red dot positioned at an impact score of approximately 0.4 and a −log10(*p*-value) of about 1.56, accompanied by other pathways depicted as smaller orange and yellow dots at lower impact and significance levels. The accompanying metabolite network diagram (Figure 6B) visualizes the structural topology of the beta-alanine metabolism pathway through a hierarchical node structure, with metabolites labeled by KEGG IDs and connected by directed arrows indicating reaction flows; central to this is beta-alanine (C00099, highlighted in red. The detailed pathway schematic (Figure 6C) maps the biochemical reactions in beta-alanine metabolism, illustrating beta-alanine as a central node derived from multiple sources including pyrimidine catabolism (uracil to dihydrouracil to 3-ureidopropionate), polyamine degradation (spermidine and spermine via 3-aminopropanal), aspartate decarboxylation (aspartate to beta-alanine), and propanoate metabolism.

## 3. Discussion

The main objective of this study was to characterize the urinary metabolic landscape of individuals across the spectrum of HTLV-1 infection, encompassing HAM, IS, ASC, HCs, while harnessing machine learning algorithms to identify discriminative metabolic features and potential biomarkers for non-invasive HAM diagnosis. Our untargeted GC-MS analysis revealed 86 significantly altered metabolites, predominantly amino acids, and their derivatives, with machine learning and ROC analyses identifying key discriminators such as Unknown_151, Unknown_127, histidine, alanine, and 4-hydroxyphenylacetic acid, which exhibited strong reductions in HAM urine and excellent classification performance (AUCs 0.855–0.871). These metabolites were selected because they consistently ranked highly in univariate statistics and multiple machine learning models, which we interpret as evidence of a robust, though still exploratory, urinary signature that requires confirmation and formal feature stability assessment in larger validation cohorts. The complexity of metabolic alterations in HAM was further demonstrated by the discrepancy between linear and non-linear modeling. While linear PLS-DA showed only modest predictive power (R^2^ = 0.44, Q^2^ = 0.23), non-linear Random Forest and SVM models achieved high diagnostic accuracy (AUC > 0.85), highlighting the importance of advanced machine-learning methods for clinical metabolomic data, and aligning with recent methodological work on AI-driven biomarker discovery [18,19]. Importantly, we confirmed that these reductions were not due to age or sex differences between the groups. Global GLM sensitivity analysis adjusting for age and sex showed that 93% (80/86) of differential metabolites, including all top discriminators such as histidine and beta-alanine, remained significant, indicating that these reductions are unlikely to be driven by demographic differences alone. These perturbations converged on enriched pathways including beta-alanine metabolism (*p* = 0.0275, impact = 0.4), phenylalanine-tyrosine-tryptophan biosynthesis (enrichment ratio > 40, *p* < 0.005), and lysine degradation, underscoring a metabolic milieu characterized by heightened protein catabolism, potential neuroprotective adaptations, and disruptions in energy cycling that may exacerbate neuroinflammatory processes. The central positioning of beta-alanine (C00099) in the pathway topology, facilitating carnosine synthesis via beta-alanyl-histidine conjugation, is consistent with the notion of a depleted neuroprotective reservoir; carnosine, a dipeptide with histidine, functions as a reactive carbonyl scavenger (e.g., quenching acrolein), pH buffer, antioxidant, and antiglycative agent, potentially attenuating excitotoxic glutamate release, ROS accumulation, and chronic inflammation in HAM [22]. We speculate that decreased beta-alanine and histidine levels could thus increase neuronal vulnerability by limiting carnosine-mediated antioxidation, a mechanism that could be further influenced by HTLV-1 Tax protein’s interference with mitochondrial function and amino acid transporters, leading to dysregulated polyamine degradation (spermidine/spermine to 3-aminopropanal) and pyrimidine catabolism (uracil to 3-ureidopropionate), both upstream sources of beta-alanine. These metabolic shifts not only corroborate the pathophysiological burden of HTLV-1 on systemic homeostasis but also reveal a cascade where viral persistence drives amino acid depletion, fostering a proinflammatory, catabolic state that bridges asymptomatic to symptomatic progression, with IS exhibiting intermediate profiles suggestive of early reprogramming as a potential early warning system.

Beyond the beta-alanine-carnosine axis, our findings indicate a broader role for oxidative stress in HAM pathophysiology. Several of the most discriminatory metabolites, including histidine, alanine, and aromatic amino acids, participate directly or indirectly in redox buffering and nitrogen balance. Their depletion in HAM suggests a reduced capacity to neutralize reactive oxygen and nitrogen species. Amino acids such as histidine and cysteine support endogenous antioxidant defenses through metal chelation, free radical scavenging, or as precursors for glutathione and carnosine. Sustained reductions in these pools may lower the threshold for oxidative damage to myelin and axons. Additionally, enrichment of pathways related to mitochondrial energy metabolism, such as the citrate cycle and glutamate metabolism, and inherited amino acid handling disorders aligns with a metabolic environment in which mitochondrial strain, protein catabolism, and redox imbalance are closely interconnected. We propose that the urinary signature observed in HAM, characterized by depleted amino acids with antioxidant or anaplerotic roles, reflects a systemic response to chronic oxidative stress driven by HTLV-1-induced inflammation, which may, in turn, exacerbate neurodegeneration in the spinal cord.

Given the novelty of this metabolomics study in HAM patients, there are few direct comparisons; however, parallels with multiple sclerosis (MS), a neurological disease that shares several similarities with HAM, offer valuable insights [23,24]. In MS, metabolomics likewise reveals dysregulations in amino acid metabolism, including histidine and beta-alanine pathways [25], which are implicated in altered energy metabolism [26,27], mitochondrial dysfunction [28], and oxidative stress [29], mirroring the metabolic fingerprint observed here. For instance, reduced histidine levels in MS serum correlate with impaired myelin repair and inflammation, akin to the diminished histidine in HAM urine that may compromise neuroprotective mechanisms [25]. Furthermore, beta-alanine’s role in MS extends to supplementation studies demonstrating potential benefits in alleviating fatigue and enhancing motor function, suggesting that the enriched beta-alanine pathway in HAM might represent a compensatory effort against neurodegeneration, though its depletion could accelerate disease progression [30,31]. Alterations in aromatic amino acid metabolism, such as phenylalanine and tryptophan, are also recurrent in MS, where they contribute to immunomodulatory deficits and metabotoxin accumulation, paralleling our observations and supporting the notion that HTLV-1-driven neuroinflammation elicits conserved metabolic responses across similar disorders [32]. This convergence argues for shared pathophysiological underpinnings, where viral persistence in HAM mimics the autoimmune triggers in MS, leading to comparable metabolic exhaustion in amino acid pools that fuel excitotoxicity and glial activation. Debates arise regarding causality: while these changes could stem from direct viral effects on host metabolism, they might alternatively reflect secondary consequences of chronic inflammation or even gut microbiota dysbiosis, as inferred from MS studies linking urinary metabolites to microbial influences [33]. Nonetheless, the robust discriminatory power of metabolites like alanine and histidine, supported by ROC analysis and machine-learning models, positions them as promising biomarkers, potentially bridging diagnostic gaps in HAM akin to how amino acid profiles aid MS monitoring.

While several top discriminators remain chemically unidentified (e.g., Unknown_151), their consistent presence across samples, distinct chromatographic peaks, and reproducible selection by multiple statistical and machine learning methods suggest they are robust metabolic features of HAM rather than technical artifacts or batch-specific noise. However, as Level 4 entities under the Metabolomics Standards Initiative, they cannot yet be considered definitive biomarkers. Future studies using higher-resolution LC-MS/MS platforms and independent validation cohorts are essential to determine their chemical identities and confirm their biological relevance.

A key strength of this study lies in its pioneering integration of untargeted urinary metabolomics with advanced machine learning in an HTLV-1 cohort, enabling the identification of subtle, yet biologically coherent, signatures in a non-invasive biofluid that is readily accessible for clinical translation. The inclusion of intermediate syndromes further enriches the dataset, capturing the disease continuum and highlighting metabolic gradients that could inform prognostic tools.

Despite these promising findings, this study has several limitations. The modest sample sizes, particularly for the IS group, stem from recruitment challenges in low-prevalence settings, potentially limiting statistical power and generalizability. Additionally, the cross-sectional design precludes causal inferences, and untargeted metabolomics, while comprehensive, may overlook low-abundance species without targeted validation. In accordance with current recommendations for clinical machine learning studies, which emphasize the risk of overly optimistic performance estimates in small cohorts and the limitations of cross-validation alone as a surrogate for out-of-sample generalizability, we note that our models were rigorously evaluated using 10-fold and repeated k-fold cross-validation with permutation testing but remain internally validated only. Although the stable AUC values (>0.85) and concordance among machine learning, univariate statistics, and pathway analyses support the internal robustness and biological plausibility of the identified urinary signature, they do not fully substitute for external validation on independent cohorts. Therefore, future studies should prioritize multi-center recruitment, including larger longitudinal cohorts, to externally validate these models across diverse populations, track metabolic trajectories, and confirm biomarkers in different ethnic groups, ideally incorporating multi-omics approaches to disentangle viral, host, and environmental contributions. In larger, externally validated cohorts, it will also be important to systematically report imbalance-aware performance metrics, such as balanced accuracy, precision–recall curves, and per-class sensitivities, particularly for HAM and IS, to fully characterize classifier behavior. Such endeavors could pave the way for personalized interventions, including hypothesis-driven strategies such as beta-alanine supplementation, inspired by MS paradigms, to test whether modulating these amino acid pathways can ameliorate HAM progression. Future studies should use multi-omics approaches (proteomics, transcriptomics) to validate the enzymatic basis of these metabolic shifts. Additionally, longitudinal monitoring of asymptomatic carriers with low beta-alanine levels could determine whether this metabolic profile predicts seroconversion to HAM.

## 4. Materials and Methods

### 4.1. Participants and Samples

The study was approved by the Institutional Review Board of the Emilio Ribas Institute (CAAE: 68008923.5.1001.0061) and the Hospital das Clínicas (CAAE: 65467022.7.0000.0068), and all participants provided written informed consent. Demographic and clinical data were collected through questionnaires and hospital electronic medical records. A total of 113 participants were included: 39 HAM patients diagnosed according to World Health Organization criteria, 17 patients with intermediate syndrome (IS), 33 asymptomatic HTLV-1-positive carriers (ASC) identified during blood donation screening, and 24 healthy HTLV-1-negative family members as controls (HC). Patients with IS were identified using the Emilio-Ribas criteria [34]. This group represents a rare but critical transitional clinical state between asymptomatic carriers and full-blown myelopathy, characterized by inflammatory symptoms and a high proviral load compared to ASCs, possibly indicating early development of HAM [35]. Although the IS group sample size is inherently limited by the difficulty of clinically capturing this transitional phase, including these patients is essential for understanding the disease’s metabolic trajectory. Exclusion criteria for HAM patients included antibiotic or probiotic use within the last four weeks, presence of other diseases, digestive disorders, tumors, or missing clinical data. For ASC and HC groups, individuals were excluded if they had a history of kidney disease, metabolic disorders (e.g., diabetes), or recent antibiotic/probiotic use. Each participant received two Norgen urine collection containers (Norgen Biotek, Thorold, ON, Canada) containing dehydrated preservative, one for morning (upon waking) and one for night (before sleeping) urine samples to capture a 24 h metabolic profile. Detailed, video-assisted instructions were provided to ensure proper sample collection and minimize contamination. Samples were transported to the laboratory by an authorized carrier and stored at −80 °C until analysis.

### 4.2. Metabolome Analysis

Urine derivatization and GC-MS analysis were performed as previously reported by Teixeira et al. [36]. Briefly, 500 µL of urine was mixed with 50 µL of DL-alanine-2,3,3,3-d4 (10 mM) as an internal standard. The sample was lyophilized for stabilization and resuspended in MilliQ water before chemical derivatization. For derivatization, 150 µL of the resuspended urine sample was alkalinized with 50 µL of 3 M NaOH, followed by the addition of 167 µL methanol and 34 µL pyridine with vigorous mixing. To derivatize primary and secondary amino groups and carboxyl groups, 20 µL of methyl chloroformate was added twice. All analyses were performed in duplicate for technical reproducibility. The derivatized samples were analyzed using an Agilent 5975C INERT XL EI/CI gas chromatograph (Agilent Technologies, Palo Alto, CA, USA) equipped with an autosampler and coupled to a single quadrupole mass detector. Separation was carried out on an HP-5MS capillary column (30 m × 0.250 mm × 0.250 μm film thickness). The oven temperature program was set as follows: initial hold at 45 °C for 2 min, ramped to 180 °C at 9 °C/min and held for 5 min, increased to 220 °C at 40 °C/min and held for 5 min, then ramped to 240 °C at 40 °C/min and held for 11.5 min, followed by a ramp to 280 °C at 40 °C/min and a final hold at 280 °C for 10 min. A 1 min post-run at 45 °C completed the cycle, resulting in a total runtime of 51 min. Helium was used as the carrier gas at a constant flow rate of 0.71 mL/min. Injections were performed in splitless mode with a volume of 1 µL, injector temperature set to 290 °C, and interface temperature at 250 °C. The mass spectrometer operated in electron impact (EI) mode at 70 eV, with the source temperature maintained at 250 °C. Data acquisition was conducted in full scan mode over a mass range of 38–550 *m*/*z*. Data acquisition and instrument control were performed using MSD ChemStation software (Version E.02.02.1431, Agilent Technologies, Palo Alto, CA, USA).

To ensure data quality, Quality Control (QC) samples were prepared by pooling equal aliquots from all urine samples. QC samples were injected at the beginning of the run to equilibrate the system and then after every 10 sample injections. Blank samples containing only reagents were analyzed to monitor carryover. Raw GC-MS data were deconvoluted and analyzed using the Automated Mass Spectral Deconvolution and Identification System (AMDIS, Version 2.71, NIST, Gaithersburg, MD, USA). Metabolite identification was performed by comparing mass spectra with the in-house MCF mass spectral library developed by the Villas-Bôas laboratory [37] and the commercially available NIST Mass Spectral Library. Identifications were classified according to the Metabolomics Standards Initiative (MSI) levels: Level 1 for high-confidence matches based on mass spectra and retention times of authentic standards, Level 2 for putative annotations, and Level 4 for unknown features. In this study, named metabolites were identified at Level 1 using the in-house library, while unmatched features were classified as Level 4. A comprehensive list of all detected metabolites and their assigned MSI identification levels is provided in Supplementary Table S1.

Peak intensities were normalized to the internal standard (DL-alanine-2,3,3,3-d4) before bioinformatic analysis to correct for technical variations in sample preparation and injection. The normalized data set was then processed using MetaboAnalyst 6.0 [38]. Missing values (zeros) were imputed by replacing them with one-fifth of the minimum positive value for each variable, assuming these values were below the limit of detection (LoD). No additional sample normalization was applied to the platform. To approximate a normal distribution and correct for heteroscedasticity, the data were log-transformed (base 10) and autoscaled before multivariate analysis. One-way analysis of variance (ANOVA) with Tukey’s post hoc test (*p* < 0.05) identified significant differences among the HAM, IS, ASC, and HC groups. Sparse Partial Least Squares Discriminant Analysis (sPLS-DA) was used to identify metabolites contributing most to group separation, with model validity assessed via permutation tests (n = 100) estimating Q^2^ and R^2^Y parameters. Key metabolites were further analyzed for pathway enrichment.

### 4.3. Machine Learning Analysis

Machine learning (ML) analysis was performed using MetaboAnalyst 6.0. Normalized and log-transformed metabolomic peak intensity data were uploaded for supervised classification. Random Forest (RF) (using 500 tree) and Support Vector Machine (SVM) algorithms were applied to discriminate HAM, ASC, and HC groups using default parameters in MetaboAnalyst 6.0. Feature importance from RF models was used to prioritize metabolites contributing to classification. Model validation employed repeated k-fold cross-validation embedded in MetaboAnalyst 6.0 to mitigate overfitting and assess model generalizability. Classification metrics, including accuracy, sensitivity, specificity, and area under the receiver operating characteristic curve (AUC-ROC), were calculated. Permutation testing (n = 100) assessed statistical significance relative to random classification. Metabolites identified as important in ML models were cross-validated using univariate statistics and subjected to pathway enrichment analysis to evaluate biological relevance. In addition to overall accuracy and ROC AUC, we examined class-specific sensitivity and specificity for HAM in the binary HAM versus non-HAM comparisons to ensure that performance was not driven solely by the larger non-HAM group, using output from MetaboAnalyst 6.0.

All models were implemented in MetaboAnalyst 6.0 using its default randomization and cross-validation routines (500-tree random forest, linear SVM, repeated k-fold cross-validation, permutation testing). Although the web interface does not allow explicit random seed settings, repeated runs with identical settings produced qualitatively identical feature rankings and performance metrics, supporting the reproducibility of the results.

### 4.4. Pathway Analysis

Pathway enrichment and impact analyses were conducted using MetaboAnalyst 6.0, utilizing the Kyoto Encyclopedia of Genes and Genomes (KEGG) database as a reference for metabolic pathways [39]. To enhance interpretability, the significantly altered candidate metabolites were divided into two categories, common (non-lipid) metabolites and lipid metabolites, and analyzed separately. The metabolite lists, annotated with their corresponding Human Metabolome Database (HMDB) identifiers, were uploaded to the platform for mapping onto reference pathways. MetaboAnalyst employs a hypergeometric test to assess the overrepresentation of metabolites within specific pathways, combined with a relative betweenness centrality measure to evaluate the pathways’ topological importance within the metabolic network. To control for multiple hypothesis testing, False Discovery Rate (FDR) correction was applied. For this study, pathways were considered statistically significant if they met either of the following criteria: (1) *p*-value < 0.05 with FDR < 0.2, or (2) *p*-value < 0.05, FDR < 0.3, and pathway impact score greater than 0.2. The significant pathways identified were visualized through interactive pathway maps, providing insights into key metabolic alterations within both metabolite categories.

### 4.5. Assessment of Confounding Factors

To ensure that the identified metabolic signatures were specific to the disease state and not influenced by demographic variables, we conducted a comprehensive assessment of confounding factors. First, we performed a global sensitivity analysis on the entire metabolomic dataset (175 metabolites) using Generalized Linear Models (GLMs) in MetaboAnalyst 6.0. We compared the *p*-values from univariate analysis (Disease) with those from a model adjusted for age and sex as covariates.

To further validate the independence of key biomarkers, we conducted Pearson correlation analyses (metabolite abundance vs. age) and Student’s t-tests (male vs. female) exclusively within the healthy control (HC) group for the top discriminative metabolites. This step distinguished physiological demographic effects from disease pathology in a non-disease context. Potential batch effects were minimized by randomizing the sample run order and normalizing data to the internal standard (DL-alanine-2,3,3,3-d4).

## 5. Conclusions

In conclusion, this study demonstrates that urinary metabolomics combined with machine learning can effectively distinguish HAM patients from non-HAM individuals, including asymptomatic carriers, intermediate syndromes, and healthy controls. The identification of dysregulated amino acid pathways, specifically beta-alanine and histidine, suggests potential therapeutic targets related to neuroprotection and oxidative stress. These findings support the development of noninvasive, urine-based diagnostic tools for HTLV-1-associated neurological disease.

## Figures and Tables

**Table 1:** Demographic and clinical characteristics of study participants.

Characteristic	HAM Patients (n = 39)	IS Patients (n = 17)	ASC Individuals (n = 33)	Healthy Controls (n = 24)
Sex (Female/Male)	28/11	15/2	25/8	12/12
Age Range (years)	37–81	38–74	28–81	18–66
Median Age (years)	57	57	60	39
Mean Age (years)	55	56	58.3	38.6
Urinary Symptoms				
- Urine Retention	15	4	N/A	N/A
- Urine Incontinence	22	7	N/A	N/A
- Both Retention and Incontinence	8	2	N/A	N/A
Diagnosis Time (years)				
- 0–5	10	N/A	N/A	N/A
- 6–10	9	N/A	N/A	N/A
- >10	20	N/A	N/A	N/A
On Treatment	25	N/A	N/A	N/A
- Oral Corticosteroids	16	N/A	N/A	N/A
- Pulse Therapy	9	N/A	N/A	N/A

Note: ASC = Asymptomatic Carriers; IS = HTLV-1-infected individuals with intermediate syndromes HAM = HTLV-1-associated myelopathy patients; HC = Healthy Controls; N/A = Not applicable.

## Data Availability

Supplementary Table S1 contains the complete list of identified metabolites together with their MSI confidence levels. The underlying raw mass spectrometry files are very large and are therefore not publicly deposited; they are available from the corresponding author upon reasonable request.

## Supplementary Materials

The following supporting information can be downloaded at: https://www.mdpi.com/article/10.3390/ijms27041827/s1.

## Abbreviations

The following abbreviations are used in this manuscript:

HTLV-1	Human T-cell lymphotropic virus type 1
HAM	HTLV-associated myelopathy patients
IS	Intermediate syndrome
ASC	Asymptomatic carriers
HC	Healthy controls
ATL	Adult T-cell leukemia/lymphoma
GC:MS	Gas chromatography:mass spectrometry
MSI	Metabolomics Standards Initiative
HMDB	Human Metabolome Database
ANOVA	Analysis of variance
PLS-DA/sPLS-DA	(Sparse) partial least squares:discriminant analysis
RF	Random Forest
SVM	Support Vector Machine
ML	Machine learning
GLM	Generalized linear model
AUC/AUC-ROC	Area under the receiver operating characteristic curve
QC	Quality control (samples)
LoD	Limit of detection
KEGG	Kyoto Encyclopedia of Genes and Genomes
FDR	False discovery rate
EC	Enzyme Commission (number)
