# Supplementary material for: Urine Metabolomics and Machine Learning Identify Metabolic Features and Potential Biomarkers of HTLV-1-Associated Myelopathy (HAM)
Source: Int J Mol Sci. 2026 Feb 14;27(4):1827. doi: 10.3390/ijms27041827 (PMC12940815; doi:10.3390/ijms27041827)
Supplement: Supplementary file 1 [file ijms-27-01827-s001.zip › Supplementary Figure S1.pdf]

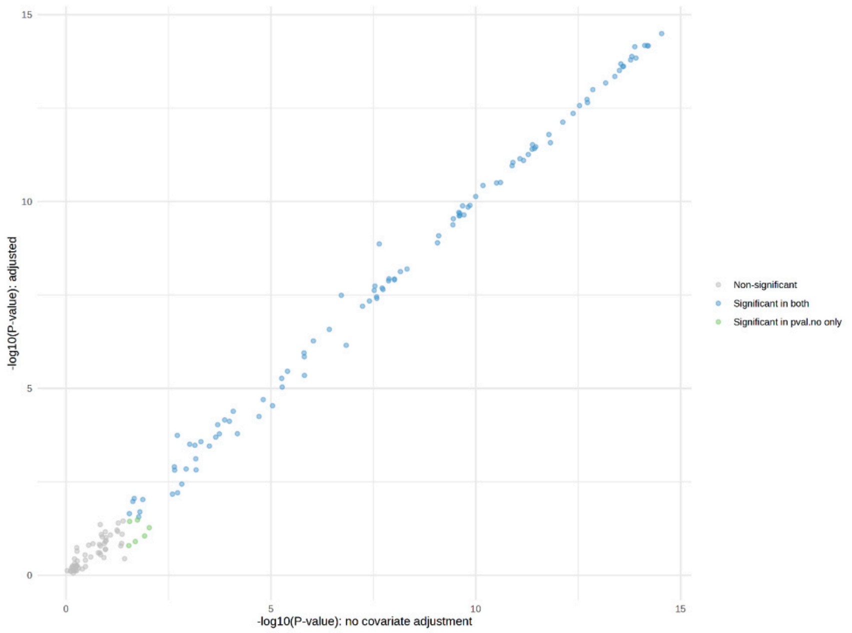

Figure S1. Global sensitivity analysis of confounding factors. A scatter plot compares the statistical significance (-log10 p-values) of all 175 detected metabolites before and after covariate adjustment. The X-axis shows significance from a standard univariate analysis (Disease Group only), while the Y-axis shows significance from a Generalized Linear Model (GLM) adjusting for age and sex as covariates. Blue dots indicate metabolites that remained statistically significant ($p < 0.05$) in both models. Green dots represent metabolites significant only in the unadjusted analysis (lost significance after correction), while gray dots represent non-significant metabolites. The strong linear correlation along the diagonal demonstrates that most metabolic alterations, including key biomarkers, are driven primarily by HTLV-1 pathology rather than demographic differences.
